# A first study on the usability and feasibility of four subtypes of suicidality in emergency mental health care

**DOI:** 10.1186/s12888-023-05374-8

**Published:** 2023-11-27

**Authors:** Remco F. P. de Winter, Connie M. Meijer, Anne T. van den Bos, Nienke Kool-Goudzwaard, John H. Enterman, Manuela A.M.L Gemen, Chani Nuij, Mirjam C. Hazewinkel, Danielle Steentjes, Gabrielle E. van Son, Derek P. de Beurs, Marieke H. de Groot

**Affiliations:** 1 Mental Health Institute Rivierduinen, GGZ Rivierduinen, Sandifortdreef 19, 2333 ZZ Leiden, The Netherlands; 2 School for Mental Health and Neuroscience (MHeNs), Maastricht University, Maastricht, The Netherlands; 3 Mental Health Institute Parnassia Group, The Hague, The Netherlands; 4 Section of Clinical Psychology, Free University (VU), Amsterdam Public Health Research Institute, Amsterdam, The Netherlands; 5 Sussex Partnership NHS Foundation Trust, Eastbourne, England; 6 University of Amsterdam, Amsterdam, The Netherlands; 7 Mental Health Institute Lentis GGZ, Groningen, The Netherlands

**Keywords:** Suicidality, Suicidal behaviour, Suicidal behavior, Differentiation of suicidality, Validation study, Suicidal subtypes, Suicidal typology, Tailored assessment, Mental health

## Abstract

**Background:**

Based on clinical experience, a (hypothetical) four-type model of suicidality that differentiates between subtypes with a unique pathway to entrapment ((h)4ME)was developed. The subtypes are: 1) perceptual disintegration (PD), 2) primary depressive cognition (PDC), 3) psychosocial turmoil (PT) and 4) inadequate communication/coping (IC). This study was carried out to examine the usability and feasibility of the subtypes in an absolute and dimensional way with the SUICIDI-2 instrument.

**Objective:**

A first step was to examine the model and the SUICIDI-2 instrument for usability and feasibility in clinical practice. We aim to investigate the’real life’ practical application of the model and hope the feedback we get after practical use of the model will help us with improvements for the model and the SUICIDI-2 instrument.

**Methods:**

Discharge letters to general practitioners of 25 cases of anonymized suicidal emergency patients were independently reviewed by three psychiatrists and three nurses. Using the SUICIDI-2 instrument, describing the proposed subtypes, cases were classified by the psychiatrists and nurses. Intraclass Correlation Coefficients (ICC) for absolute/discrete and dimensional ratings were calculated to examine the model’s usability and the instrument‘s feasibility. The study was approved by the ethical board.

**Results:**

All raters were able to recognize and classify the cases in subtypes. We found an average measure of good reliability for absolute/(discrete) subtypes. For dimensional scores, we found excellent average measures for the subtype PDC, and good average measures for the subtypes PD, PT and IC. The reliability of dimensional score for the SUICIDI-2 was relatively lower than an alternative dimensional rating, but had good ICC values for all subtypes. After reviewing the results though, we found some inconsistently assessment between raters. This was ground to narrow down the criteria per subtype to describe the subtypes more precisely. This resulted in adjusted formulations for subtypes PD and IC and agreement was achieved about formulations in the revised SUICIDI-3.

**Conclusions:**

The hypothetical model of entrapment leading to suicidality shows promising results for both the usability and feasibility of the SUICIDI instrument. Follow up studies with participants with a more diverse background may show consistency and validity for the model.

## Background

Suicidality includes suicidal ideation, plans and suicide attempts. It is considered a calamity in mental health care, general health care and in general society. In developed countries, the prevalence of suicidal ideation, plans and attempts in the adult population over 12 months is 2.1%, 0.7%, 0.4% respectively [1]. Suicidality is complex and multifactorial, and the result of a wide range of interacting psychological, psychiatric, genetic, social, economic and cultural, risk factors operating at multiple levels (societal, community, relationship, and individual) [1]. It is an erratic pattern of thoughts and behaviours that serves as a precursor to suicide. Suicide is the leading cause of non-natural death worldwide and the second leading cause of mortality in individuals aged 15–29 years [2]. Suicide is widely considered the worst possible outcome within mental health care.

Suicidality is a heterogeneous, seemingly non-consistent phenomenon [3] and it is not a clear-defined psychiatric symptom. Officially, it only occurs as a symptom in two psychiatric classifications according to the ‘Diagnostic and Statistical Manual of Mental Disorders’ (DSM-5): major depressive disorder and borderline personality disorder [4, 5]. For a number of psychiatric diagnoses though, suicidality is a frequently occurring symptom as is the case for PTSD, sleep disorders and adjustment disorders [6, 7]. Despite the great complexity involved in the assessment and risk assessment of suicidality, there is little empirical research on the differentiation of subtypes of this phenomenon [8, 9]. Guidelines tend to focus on general aspects of the assessment and treatment of suicidality, but, apart from making a distinction between an acute and chronic type, a clear differentiation of suicidality is lacking [10–12].

To date, management of the various ways of expressing suicidality is based on knowledge of risk factors, clinical experience and partly and probably on intuition. Crisis services and acute admission wards are frequently confronted with serious suicidality and make a significant contribution to the prevention of suicide [13, 14]. Outreaching psychiatric emergency services become involved in assessment of suicidality when it is regarded -or suspected- as a critical event by the community, the patient themselves, significant others and healthcare professionals. Acute emergency services are required to set up policies around suicidality, appropriate treatment and safety planning [15]. In the Netherlands, the employees of these services are almost exclusively medical doctors or specialized consultant psychiatrists and (specialised) nurses. Psychologists are rarely present within these services [16]. The presence of stress factors, vulnerability factors and protecting factors are weighed to estimate the suicide risk and establish the consequent management strategy. An independent, consultant psychiatrist decides which policy will be applied, based on the assessment of the crisis service*,* for example whether or not a patient will be voluntarily or involuntarily moved to a psychiatric emergency facility [17].

**Fig. 1:**
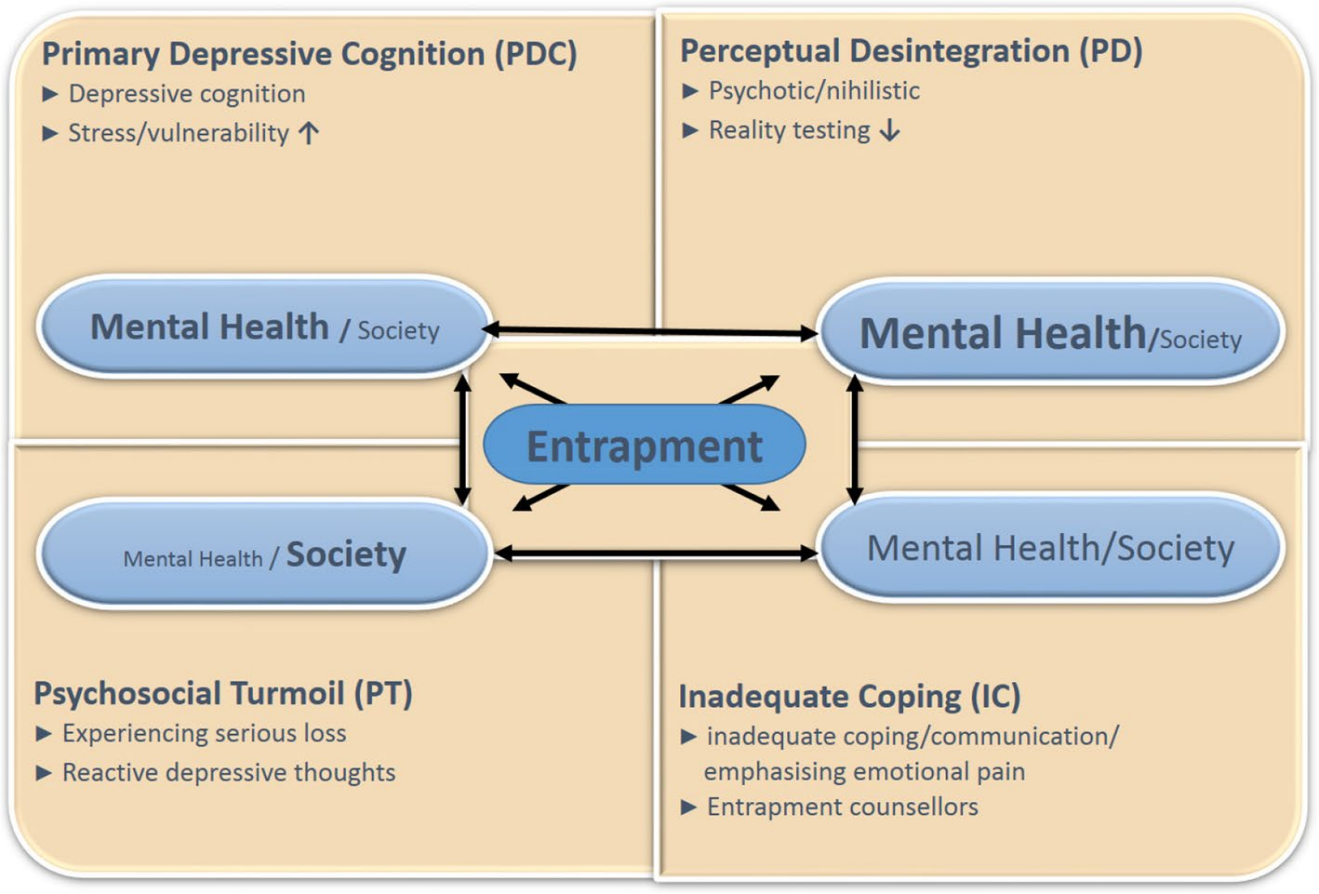
Hypothetical model for 4 suicidal subtypes. Degree of responsibility for (mental) health (MH) care or patient with society (community) [3]

The experience of entrapment plays a crucial role in suicidality as described in the integrated model of stress vulnerability [18] and entrapment model [19], developed for the Dutch multidisciplinary guideline on suicide and suicidal behaviour [20]. Entrapment is described as the perception of being trapped: the stronger the feeling of entrapment, the higher the risk of suicidal acts [19]. In some cases, it is unclear or not established to what extent (mental) health care workers are responsible to prevent escalation of suicidal behaviours. Differentiation of the entrapment etiology may be helpful to determine this [3] (Fig. 1).

Although the current knowledge of suicidality has not yet reached the point at which we know and understand the exact mechanisms behind the development of suicidal conditions. We believe that differentiation of entrapment leading to suicidality will support improved clinical and practice through better risk assessment and prognosis. There are evidence-based strategies to prevent (attempted) suicide. It would be helpful to gain insight into which (existing) prevention or recovery strategy is (more) effective and for who. Differentiation of suicidality may allow accurate scientific research. Finally, formulation of different levels of (shared) responsibility of (mental) health workers and patients may be improved [21–24].

So far, we have developed a (hypothetical) 4-type model of suicidality ((h)4ME), differentiating four pathways to entrapment [12]. The (h)4ME [3] is based on both clinical practice [3] and on a theoretical dimensional approach of psychopathology and personality [25]. Previously, this (hypothetical) 4-type model of suicidality is extensively discussed with colleagues and patient experts at several conventions, including a discussion forum in which 50 psychiatrists took part [3, 26]. Subsequently, the model was revised accordingly. To be able to investigate the model’s usability the SUICIdality DIfferentiation(SUICIDI-2) [3] instrument was developed and updated over the recent years. See this link https://suicidaliteit.nl/SUICIDI/SUICIDI%20translation.pdf. 

The four types of entrapment etiology are: [3] (Fig. 1):

1. Perceptual disintegration (PD); originated in the context of disturbed perceptions and/or behaviours,
2. Primary depressive cognition (PDC); in the context of (a) depressive cognition(s),
3. Psychosocial ‘turmoil’ (PT); originated in the context of acute reactivity to a (deemed or actual) loss, offence, adversity or doom,
4. Inadequate communication/coping (IC) (emphasizing emotional pain); originated in the context of communicating about intense suffering.

See Table 1 for a more detailed description of the subtypes as described before [3].

**Table 1:** Descriptions of the four subtypes of suicidality. (Table 2 in de Winter et al. 2023 [3])

**Perceptual Disintegration (psychotic disturbed perception/behaviour) (PD)** Suicidality originates from psychosis, which can often be accompanied by affective (depressive) dysregulation or can be affected by it. Usually, the psychotic state has only been present for probably a short time (rather days or weeks than months) and is noticed (or becomes apparent) because of its severity. Suicidality may originate from depressogenic cognition; however, in that case, the severity has developed to such a level that it can be seen as a mood-congruent or mood-incongruent psychotic state. The distress can be understood, but the severity cannot be perceived as comprehensible anymore by the examiner. A classic state is a depression with mood-congruent psychotic features. However, it can also appear among people who, while in a psychotic state, are ordered by their delusions to hurt themselves
**Primary Depressive Cognition (PDC)** Suicidality stems primarily from a depressive thought process and there are no psychotic features (yet). The depressive state can be present for a while (eg, weeks or months). Thoughts of suicide, which are part of the cognition and present on a daily basis, are characteristic. There is clear evidence of distress, which can be noticed by the examiner because of the depressive thought process. A classic example would be a depressive disorder, but primary depressive cognition may also be part of an anxiety disorder, autism, etc. The features of a personality disorder may be mixed with the depressive state, or the depressive state may be caused by a personality disorder and become part of a returning thought pattern in which negative cognitions and Beck cognitive triad can be present (negative views about oneself, negative views about the world, and negative views about the future)
**Psychosocial Turmoil (PT)** Suicidality stems primarily from a severe loss or blow to the ego, leading to a complete upheaval of someone’s life. The person experiences enormous guilt, severe shame, or does not dare to look another in the eye anymore or experiences a downfall without being in a psychotic state. There is an unbearable anguish, which leads to a need for release from that pain or the need not to exist anymore, to not be able to feel or escape the awful misery or pending dread. Usually, someone has been in this state for a short time (hours, days, or weeks). Drug use can be extra provoking. The stress is perceivable for the examiner from the perspective of loss or a blow to the ego and there may be slight psychotic features, but one can follow the narrative. Underlying dysregulation of the impulsivity can worsen the state and increase the risk of a lethal outcome
**Inadequate Coping/communication (IC)** Suicidality stems from a severe feeling of distress and not being able to communicate this properly. There is difficulty with formulating an adequate request for help and one seems to be hoping for a solution by demonstrating suicidality. This behaviour usually exists for a longer period (months) and fluctuates severely. This type of a more chronic suicidality is often seen as part of a personality disorder such as a borderline personality disorder. Also, drug use can be an important provoking factor. Suicidality is perceived by others as “externalizing” and fake and can result in aid workers feeling “trapped” in the dynamics. The behaviour can coincide with experiences of loss with which the powerlessness is externalized and not internalized. Often, the support system is exhausted and professionals are viewed as failing. The major risk is for professionals to feel manipulated, and for the person who is assessed to feel misunderstood and not taken seriously, which leads to an amplification of the behaviour, accompanied by an increased risk of suicide. Contrary to how it is perceived by others, the person is genuinely in distress. Suicide can be used as the ultimate way to communicate about the distress caused by the perceived unfair or rejection judgment of the person (especially recognizing and exploring the countertransference and offering help to the underlying motivators of suicidality are essential with this type)

The study protocol is previously described [3]. In this study, we aim at examining the usability of the (hypothetical) 4 type ME and the feasibility of the SUICIDE-2 [3]. We aim to answer the following questions:

1. Is the differentiation model practically useful for a selection of mental health care workers?
2. Can conclusions of patient records of suicidal high risk patients assessed by the outreach psychiatric emergency services, be rated in an absolute/discrete and dimensional way?
3. Can clinicians allocate cases to the proposed subtypes (PD, PDC, PT and IC)?
4. How are subtypes distributed or subdivided across the group?
5. Are these subtypes dimensionally delineated by using two different modes of gradual rating (SUICIDI instrument and 0–4 score) and is there consensus when different clinicians/investigators independently score them? What is the reliability of the different modes of rating?
6. What mode of dimensional rating is preferred in future research? Is there any need to adjust the SUICIDI-2 instrument?
7. What feedback can we provide to raters when there is any indication that raters rated incorrectly?

## Methods

### Design

This is a quantitative and qualitative exploration study [3]. In the current study, expert mental health workers were asked to allocate anonymised case descriptions of suicidal patients to subtypes of entrapment. The recruitment procedure for raters was described before [3]. The profession of the raters was matched as closely as possible with the professions represented in the outreaching psychiatric emergency service. The characteristics of the raters are described in Table 2. The raters (CM, AvdB, JE, NK, MG, MdG) were found in the collegial and scientific network of RdW over four mental Health institutions; three in The Netherlands and one in England. Three Zoom sessions were held with RdW and the six raters (February, March and June 2021) in which the (h)4ME model was explained and detailed instructions for correct rating were provided. Rating forms and summarized details of cases to be rated were sent on June the 30^th^ 2021. The deadline for submitting ratings was September 1^st^ 2021*.*

**Table 2:** Characteristics of raters and scoring procedure

*Rater*	*profession*	*Experience (years)*	*Practising in mental health*	*Institute*
*1*	Nurse scientist Ph.D	40	V	^Lentis^
*2*	Nurse scientist Ph.D	35	X	^Parnassia^
*3*	Nurse BSc	45	V	^Rivierduinen^
*4*	Psychiatrist MD	38	V	^Parnassia^
*5*	Psychiatrist MD	36	V	^NHS^
*6*	Psychiatrist MD	20	V	^Rivierduinen^
***all***	Scoring	***Absolute/discrete score***	***Gradual SUICIDI***	***Gradual 0–4 score***
	***Subtype***			
	*PD*	Yes/no	0–2	0–4
	*PDC*	Yes/no	0–2	0–4
	*PT*	Yes/no	0–2	0–4
	*IC*	Yes/no	0–2	0–4
	**Scoring**	**Only one time yes**	**Score per subtype**	**Always 4 points**

### Participants and data collection

Discharge letters to general practitioners of 25 cases of anonymized suicidal patients were independently reviewed by three psychiatrists and three nurses (raters). Using the SUICIDI-2 instrument describing the proposed subtypes, cases were classified by the raters.

Participants are suicidal patients (*n* = 25) assessed by the The Hague outreaching psychiatric emergency service [3]. Under supervision of RdW a detailed report of every assessment was jointly produced by a medical doctor and a mental health nurse, and the reports were supervised and discussed by consultant psychiatrist RdW. All assessments were discussed and evaluated in the morning hand-over by a team of at least five mental health care workers.

Of every case, an anonymized conclusion was prepared for the raters (see also Table 3). A total of 503 cases were included in a database. Only patients who consented to the discharge letter, signed by RdW, being sent to their general practitioner and who consented that information for compliant with legal standards of privacy and patient confidentiality was exchanged, were included. For this study, we included the first 25 individual cases (no duplication due to subsequent assessments of one patient) between January 2018—March 2018. Patients identities were safeguarded through case coding, while details such as gender, age, marital status and cultural background were documented. The DSM-5 classification [5] was used to establish the primary diagnosis and for eventual additional classifications. Cases entered the database after the first assessment.

**Table 3:** All absolute scores for all 6 raters

Case	Perceptual	Depression	Turmoil	Coping	Points	Classification (other secondary classification (s),—is none)
1	1		4	1	6	AS (DD)
2		**6**			**6**	**DD (AD)**
3		1		5	6	AS (PSD, DD)
4		5	1		6	DD (-)
5		**6**			**6**	**PTSD (ED)**
6		2	1	3	6	PSD (AS, PTSD)
7	1	1		4	6	BD (-)
8	1	1	2	2	6	AS (-)
9			**6**		**6**	**AS (DD)**
10		5		1	6	DD (AD(H)D)
11	1	1	1	3	6	PTSD (-)
12	2	2	2		6	DD (-)
13		**6**			**6**	**BD (AS)**
14			2	4	6	DD (AS)
15			4	2	6	AD(H)D (-)
16		**6**			**6**	**DD (-)**
17				**6**	**6**	**PD (ASD)**
18			2	4	6	AS (PTSD)
19			5	1	6	AS (-)
20	**6**				**6**	**PD (-)**
21			4	2	6	PSD (AS)
22	1	1		4	6	ED (PSD)
23				**6**	**6**	**PD (AS)**
24		2		4	6	DD (ED)
25		5		1	6	ASD (PSD)
Total	13 (8.7%)	50 (33%)	34 (22.7%)	53 (35.3%)	150	

Primary classification (abbreviation) and eventual secondary classification (abbreviation)

*Abbreviations: DD* Depressive Disorder*, AS* Alcohol/Substance abuse*, PTSD* Post-Traumatic Stress Disorder*, PD* Psychotic Disorder*, BD* Bipolar disorder*, AD(H)D* Attention-Deficit (Hyperactivity) Disorder *PSD* PerSonality disorder*, ASD* Autism Spectrum Disorder*, ED* Eating Disorder*, AD* Anxiety Disorder

The following definition of suicidality was used: “behaviours including suicidal thoughts, suicide plans, suicide attempts and completed suicide”. The definition used for attempted suicide was: "Any non-fatal suicidal behaviour, such as intentional self-poisoning, self-injury or self-harm which may or may not have a fatal intent or outcome" [27] (p. 12). We only included patients after an attempt if they were deemed suicidal. For example: if someone had cut themselves out of total despair with suicidal entrapment, this patient was included. Patients who self-harmed for a different reason without being suicidal were excluded.

### Procedure

All raters received 25 anonymized conclusions (Table 4) taken from the discharge letters to the patients GP and were asked to investigate the conclusions and to record the ratings in a prepared Microsoft ‘Excel file’ that could be transferred into ‘SPSS’ (SPSS Inc. Chicago, IL). They were asked to make an absolute choice for a discrete subtype (PD, PDC, PT or IC) for each case and even when in doubt, still choose only one option also named absolute Type Agreement (aTA). In addition, the SUICIDI-2 instrument had to be scored as a dimensional rating per subtype. With the instrument, it is possible to rate subtypes with a zero, a 1 or a 2 also named ‘dimensional Type Agreement 2’ (d2TA) see this link for the instrument. Theoretically, multiple subtypes can be rated, and there is no minimum or maximum and the total rate per case, over different subtypes, can theoretically add up to totals between 1 and more than 4 (see Table 2).

**Table 4:** Perfect cases: gives examples of a selection of 4 of the 8 perfect cases (100% consensus see also Table 3)

**Casus 20 (PD)** (Home) assessment of suicide risk concerning a 20–24 year old, Muslim woman living with her parents. She presented for assessment after she threatened to cut herself, holding a knife. Mother stopped her from doing so and the police were called. Patient completed higher level education recently and had been working in the library for a week while at the same time a beloved uncle had died. Patient seemed to have functioned normally up to a few days prior to presentation and had since become anxious and paranoid. There is no history of substance abuse and she refused to comply with somatic investigations with her GP. We saw a woman who was lying in bed underneath the covers in a darkened room, during the day, and hardly answered (open) questions. It was not clear if she would not or did not want to answer the questions. According to the information of the family, the presentation is suspect of a first psychotic episode with paranoia whilst it is not clear what the context would be. Additionally, we saw symptoms of catatonia with mutism, negativism, staring and evidence of reduced food & fluid intake. Patient was admitted involuntarily
**Casus 2 (PDC)** Suicide risk assessment of a 45–49-year-old, married mother with 3 children who presented to her GP because she was concerned about not being able to resist longstanding suicidal ideation. Suicidal thoughts had been present for approximately 3 weeks and she was not aware of any triggers. In the past she was seen once by the community team for a moderate depression but refused treatment. We saw a restless, anxious woman who could not make a reliable safety plan. As a differential diagnosis we considered an anxiety disorder (GAD with symptoms of depression) or a depressive illness with secondary anxiety. Patient had not informed anyone close to her about her symptoms and initially did not want her husband to be called. In the end she agreed for him to be informed and after the arrival of her partner she had calmed down already and a reliable safety plan could be agreed. She agreed to be followed up by the community team (acute care) and admission was avoided
**Casus 9 (PT)** Assessment of suicide risk of a 15–19-year-old, well kempt woman without a psychiatric history who presented trough the police, after she -under the influence of alcohol- jumped in front of a car after leaving a friend’s party, resulting in her being hit though not wounded. We saw a calm, friendly girl, denying suicidality, and feeling sorry and embarrassed about what happened. Sexually explicit recordings of her with several men had been distributed. Behaviour was explained by the effects of alcohol and being informed about the recordings and consequent shock. Patient is able to agree to a safety plan and has plans for the future. There are no symptoms of any underlying depression, there is no history of suicide attempts or self-harm. She goes home with her mother. Suicide risk does not appear to be acutely increased. It was decided to refer patient to suicidality aftercare care project (SUNA)
**Casus 23 (IC)** Suicide assessment of a 60–64 year old male, with a previous diagnosis of schizophrenia and gambling addiction, being under the care of the community mental health team. On the day of assessment, he had been discharged from the supported living accommodation. The decision to discharge him had been agreed by the higher management and could not be reversed. Patient had not complied with agreements, and for some time already there had been problems with aggression and being a nuisance to his environment. There had been a number of warnings and meetings with the patient about his behaviour. Patient went to “sheltered housing” but did not want to share a room with others and went on to express suicidal ideas. When seen there was no evidence of psychosis, nor was there evidence of burnt-out schizophrenia affecting his behaviour. There is no history of suicide attempts, and suicidal behaviour seems to be a lever to get what he wants, this idea being supported by the information from staff of supported accommodation and his therapist. There is no indication for admission

Between brackets the choice for the perfect cases:

I = PD

II = PDC

III = PT and

IV = IC)

As an alternative way of dimensional (gradual) scoring, raters could score a total of 4 points for the ‘Dimensional Type Agreement 1’ (d1TA) for each case, and divide these 4 points between the 4 subtypes. In theory, it was possible to award each subtype between 1 and 4 points; in the latter case leaving no remaining points to be rated for the other subtypes. In September and October 2021 a ZOOM feedback meeting for the first study and follow-up was planned. In this meeting the findings were presented, a qualitative feedback was formulated and explanations for improvement and optimizing the SUICIDI-2 instrument given.

### Ethical considerations

Before starting the study all experimental protocols were approved by the research committee of the Mental Health Institute Parnassia Group. The study adhered to relevant guidelines and regulations throughout the research process. For this study, we utilized discharge letters addressed to the general practitioner (GP) and patient data that fell under the treatment responsibility of the primary author RdW. Data collection was contingent upon obtaining verbal consent from each patient, allowing for the sharing of assessment information with the GP and the exchange of medical data. If a patient declined permission, no information was used or collected for the study. Explicit informed consent was not specifically obtained from the patients for this study. However, to ensure ethical considerations, we sought the review of the ‘Medical Research Ethics Committee Leiden-The Hague-Delft’. The committee assessed the permissibility of using and anonymizing the data in a manner that prevents the identification of individual cases. The ‘Medical Research Ethics Committee Leiden-The Hague-Delft’, in accordance with the Involving Human Subjects Act (WMO), approved the study and waived the requirement for written informed consent (G21.021/PV/pv).

### Statistical analyses

Intraclass correlation coefficient (ICC) estimates and 95% confidential intervals were calculated using ‘SPSS statistical package version 27’ (SPSS Inc. Chicago, IL) based on mean rating (K = 6). For absolute/discrete agreement we used a 2-way mixed-effects model according to the guideline for selecting and reporting ICC from Koo and Li [28]. The average values from six raters are presented. ICC values less than 0.5, between 0.5 and 0.75, between 0.75 and 0.90, and > 0.90, indicate poor, moderate, good, and excellent reliability [28] respectively.

## Results

All raters were able to use the written conclusions as provided to rate the subtypes in a dimensional and discrete manner, using the SUICIDI-2 instrument. Table 3 describes the absolute/discrete ratings for the 25 cases for all raters. For 8 cases (32%) there was 100% consensus, for 21 cases (84%) there was more than 66.6% consensus. As the absolute choice of one specific subtype, PD was chosen in 8.7% of the cases by all raters, PDC was chosen in 33% of the cases by the raters, PT was chosen in 22.7% by the raters and finally IC was chosen in 35.3% of the cases by the raters.

Table 4 gives examples of a selection of four conclusions of cases investigated by the raters with 100% consensus for each of the subtypes. For example: for case 20 every rater chose PD, for case 2 every rater chose PDC, for case 9 every rater PT and for case 23 every rater chose IC. The only 4 cases (case 6, 8,11 and 12) with less than 66.7% consensus are presented as non-perfect cases in Table 5.

**Table 5:** Non-perfect cases. Description of the 4 (non-perfect) cases with less than 66.6% consensus

**Casus 6 (≤ 0.5) (IC)** Assessment of a 40–44 year old Dutch woman with a diagnosis of PTSD, dependence on cocaine, borderline personality disorder, a history of prostitution and suicide attempts. Patient lives in sheltered accommodation and is followed up by the community mental health team. She presented at the A&E department after an overdose of 20 tablets of oxazepam 50 mg and cocaine (worth 390 euros). We assessed a desperate woman who states to be tired of life and wanting to end her horrible existence. There seems to be no end to her misery and she does not know how to proceed. She indicates she will do another suicide attempt with oxazepam if we let her go because everything is useless. She regrets the failed attempt. Ultimately, she agrees to a voluntary admission to a crisis unit to avert suicide
**Casus 8 (≤ 0.5) (?)** Assessment of a 50–54 male, known to be alcohol dependent. Presentation is triggered by an argument with his wife and son, and he made suicidal statements under the influence of alcohol. The police were informed by the neighbours. We assessed a reasonably kempt man who states that his problems stem from financial and relationship problems. During assessment alcohol abuse seems to be paramount and it makes him impulsive, and there is no evidence of current suicidal ideation or plans. He feels his support system and people close to him do not understand him, though is feeling better now. Acute suicide risk is considered not to be increased anymore. Patient says not to want help anymore and wants to be discharged so he can work his shift in a restaurant
**Casus 11 (≤ 0.5) (IC)** Assessment of suicide risk of a 30–35-year-old woman with previous diagnosis of PTSD and a dissociative disorder, known to different community teams though treatment seems to stagnate after a short period because of non-attendance to appointments. Patient was referred because of a suicide attempt by ingesting 30 tablets of peppermint oil and 30–40 tablets of diazepam 5 mg, after which she called her father to say goodbye; following this an ambulance was called. During the assessment patient states she is desperate because she has been suffering for 14 years with abdominal pain of unknown origin. Her abdominal pain dominates her life, and somatic delusions cannot be excluded. She makes a tired impression and appears desperate. Initially she says she will try to kill herself again but during the course of the assessment and involvement of her family, a safe situation is created. She also has plans for the coming week. Suicide risk is assessed as not acutely increased, and an urgent referral to the mental health community team is arranged
**Casus 12 (≤ 0.5) (?)** Assessment of suicide risk at the A&E department of a 45–49 year old man with no previous psychiatric history. He apparently referred himself to a different mental health trust and had a first meeting with them already. Patient was found by his girlfriend at home after a suicide attempt by ingesting medication (25–29 tablets containing a benzodiazepine) and pulling a plastic bag over his head, after writing farewell letters. He was transported by ambulance to A&E. There have been several experiences of loss, and his daughter attempted suicide by jumping out of the window of the family home, later stating she did not regret the attempt. Patient appears to be suffering from a low mood and is preoccupied with his financial situation (differential diagnosis is delusion of poverty). Patient believes nothing will ever be right again and he is the culprit of all misery. He perceives himself to be rotten to the core hence his daughter not being able to do anything but die. He is persistent in his wish to die and a diagnosis of severe depression with psychotic symptoms is considered. Despite an involuntary admission being regarded, he agrees to a voluntary admission. Suicide risk is assessed as acutely increased

Between brackets the choice for the non-perfect cases (case 6 and 11 IC most common, for case 8 and 12 no choice could be made by equal weight see also Table 3)

Table 6 gives the ICC for all the subtypes. Generally, reliability for every subtype (aTA) was good (95% CI: between moderate –excellent). Regarding absolute scores: PD showed good reliability (95% CI: between moderate –excellent) on average. Absolute PDC showed an average of excellent reliability (95% CI: between good–excellent). Absolute PT showed an average reliability (95% CI: between moderate-excellent) and finally, absolute IC showed good reliability (95% CI: between moderate –excellent) on average. For the dimensional scores 0–4 (d1TA) PD showed an average of good reliability (95% CI: between moderate –excellent). PDC showed an average of excellent reliability (95% CI: between good–excellent). PT showed an average of good reliability (95% CI: between good–excellent). Finally IC showed an average of good reliability (95% CI: between moderate –excellent). The reliability of the SUICIDI-2 score (d2TA) was relatively lower but gave only a lower reliability for IC (95% CI: between moderate –good). In general the ICC scores for the SUICIDI-2 instrument were lower than for the (d2TA) 0–4 score.

**Table 6:** Intraclass correlation absolute agreement coefficient Values less than 0.5 are indicative as poor reliability, moderate reliability scores between 0.5 and 0.75, good reliability for scores between 0.75 and 0.9, and excellent reliability for scores greater than 0.90

*Average measure*	*ICC*	*95% CI lower bound*	*95% CI upper bound*	*Value*	*Cronbach Alpha*
*All types (dichotomous score)*	,854	,743	,927	7,795	,872
*Absolute Perceptual (PD)*	.836	..713	.918	6.930	.844
*Absolute Depressive (PDC)*	.913	.848	.957	11.861	.916
*Absolute Turmoil (PT)*	.821	.683	.911	5.436	.816
*Absolute Communication (IC)*	.820	.586	.910	6.000	.823
*Dimensional score (0–4)*					
*Perceptual (PD) TA*	,834	,710	,917	6,478	,846
*Depressive (PDC) TA*	,932	,880	,966	14,70	,932
*Turmoil (PT) TA*	,892	,809	,946	9,992	,932
*Communication (IC) TA*	,823	,690	,912	6,327	,842
*Dimensional score SUICIDI questionnaire (0–2)*					
*Perceptual (PD) SUICIDI*	,802	,654	,901	5,535	,819
*Depressive (PDC) SUICIDI*	,871	,774	,936	8,447	,882
*Turmoil (PT) SUICIDI*	,851	,740	,926	7,328	,864
*Communication (IC) SUICIDI*	,790	,634	,895	5,150	,806

Table 7 describes the primary diagnosis. Depressive disorder and substance use was most common among the suicidal patients.

**Table 7:** Major primary DSM classifications for all suicidal patients

Major diagnosis (abbreviation)	n (percentage)
Depressive disorder (DD)	7 (28%)
Alcohol/substance abuse (AS)	6 (24%)
Psychotic disorder (PD)	3 (12%)
Personality disorder (PSD)	2 (8%)
Bipolar disorder (BD)	2 (8%)
PTSD (PTSD)	2 (8%)
ADHD (ADHD)	1 (4%)
ASS (ASS)	1 (4%)
Eating disorder (ED)	1 (4%)
*Total*	*25 (100%)*

Table 8 describes some characteristics of the suicidal patients.

**Table 8:** Description of selected characteristics of the suicidal patients

Topic	N (percentage) or mean (SD)
Actual in treatment	**10 (40%)**
Policy: IHT or admission	**10 (44%)**
Involuntary admission	**2 (8%)**
Female	**15 (60%)**
Out of office time	**11 (44%)**
Attempt	**16 (64%)**
Dutch ethnicity	**16 (64%)**
Age	**38.6 (14.6)**

During the course of the evaluation, it was noticed one of the raters had rated PD relatively often. According to this rater, alcohol and substance abuse distort perception and assessment of the situation a person finds himself in and may affect the suicidal process/suicidality. This was discussed with the raters during a follow-up session. Nevertheless, the underlying etiological basis of suicidality always prevails. It is important to look at the most common undifferentiated etiological basis causing a deregulation of the process, leading to a suicidal crisis or suicidality in general. It is explained in the model that the underlying etiological basis of the suicidality should always be found. There was some discussion about IC, which according to two of the raters was a semantic discussion about communication and the underlying process. It was explained that in case of affective dysregulation, PDC should be scored more frequently.

## Discussion

Development of subtypes of entrapment leading to suicidality was clinically motivated in order to get a better grip on a more rationalised diagnostic formulation of suicidality. Probably, many clinicians already distinguish subtypes of suicidality, but systematic knowledge of differentiation of suicidality is scarce (10). As far as we know, this is the first study investigating the usability of a differentiation model of suicidality. Results of the current study suggest that independent raters show strong consensus when they are asked to assign cases of suicidality to one or more hypothetical subtypes of entrapment as formulated in reference of the (h)4ME model using the SUICIDI-2. The SUICIDI-2 is a tool that was designed to enable clinicians to allocate cases of suicidality to one (or more) subtypes.

Because of the current and most up-to-date results, and better results with a rating of 0–4 rather than 0–2, combined with a better dimensional type agreement, the SUICIDI-2 instrument is rewritten to fit with a 0–4 rating system and the 0–2 rating was abolished, also for future research. Rewritten criteria for the SUICIDI-2 instrument are, by virtue of these results, combined with the better d1TA score and will be reformulated in a 0–4 score. The 0–2 score (d2TA) will be no longer used in future research. A revised SUICIDI-3 instrument (version 3.1) has already been made and can be found on https://suicidaliteit.nl see this link. https://suicidaliteit.nl/2023/SUICIDI/EnglishSUICIDI-3.2.pdf.

### Limitations

Because the conclusions and summaries in discharge letters were written by a nurse and a junior doctor and supervised by a psychiatrist (RdW), it is possible that the subjective clinical opinion of the clinicians was expressed in the conclusion; also because RdW developed the subtype model and was their supervisor. This may have caused bias. The sample size is small; therefore outcomes should be considered in this context. The results show that there are a few cases meeting criteria for various subtypes. So, cases may cover various subtypes, but it is unclear how coverage is distributed over subtypes [12]. It may well be possible for certain subtypes to be composed of several mixtures and a dimensional approach for subtyping instead of categorical subtypes preferred. Perhaps subtypes themselves allow further subdivision.

In addition, raters had to choose one or more subtypes as described in the SUICIDI 2; this may have led to overestimated outcomes. Suicidal patients requiring assessment by emergency services probably differ from patients being presented in outpatient community services. The current population also differs from the group of patients being admitted to a mental health ward as some types of suicidality stand out more and require a more rapid response from services. We need to consider the fact that the majority of suicides happens without intervention of mental health care services. Research into these groups seems more difficult, however it may be possible to do in follow-up studies. The model might be useful for psychological autopsies of people who did not access specialised care and for looking at whether there is a difference between subdivisions/differentiation of suicides within services and suicides outside mental health services.

Further it is important for psychologists and other mental health workers -other than psychiatrists and nurses- to gain experience with the model and to contribute to validation studies of the model. A multi-disciplinary team (psychologists, GP’s, and other related disciplines) ideally needs to be included in future research. Future research in larger and more varied samples of subjects (youth, elderly, substance abusers) and other raters than in the current study may reveal how overlap is distributed and whether subtypes are consistent over time, or whether further (sub)differentiation of types or adding subtypes are needed.

Furthermore, we are aware of the risk that rating becomes inconsistent if the system is not properly explained. We realize the importance of clearly explaining the role of the underlying ‘aetiology of entrapment’ to raters. This importance is evident in for example the case of a person with gambling debts leading to unemployment, and their partner not being aware of it. Should this person get drunk, the underlying stress will exacerbate the suicidal process and -because of the intoxication- may be perceived by a rater as perceptual disintegration. However, in this case psychosocial turmoil (PT), induced by alcohol abuse is the actual underlying trigger for the onset of entrapment [29]. As clinicians we encounter substance (such as psychedelics) use being probably responsible for suicidality at an individual level, however we cannot draw conclusions about the group in general [30]. There is no clear and unambiguous evidence for substances to be linked to suicidality. This might be investigated in future research.

### Continuation of the discussion

The differentiation in subtypes may contribute to the refinement of various factors in psychiatric research on suicidality by enhancing the differentiation of groups. This enhancement could signify the emergence of distinct cultural, economic, psychological, psychosocial, biological dysregulation, or underlying biological/genetic vulnerabilities [31–33].

It is also important to investigate which clinical and demographic features are associated with subtypes, to achieve more reliable identification of subtypes. We conducted a comprehensive assessment of suicidality in crisis situations and discerned a range of distinct manifestations that demand tailored interventions. This approach is congruent with the concept of a more refined crisis intervention, as advocated by Seguin & Chawky [34]. In a previous paper [3], we discussed the underlying etiological factors of suicidality such as perceived burdensomeness and thwarted belongingness [35, 36]. Further investigation is needed to determine whether these concepts are linked to various clinical subtypes of suicidality.

We also propose two hypothetical patterns that may better characterize chronic underlying suicidality: PDC and IC. Clinical observations suggest that the latter may, at times, manifest as an acute suicidal state within a pre-existing, long-standing chronic suicidal context. Additionally, we introduce two hypothetical patterns associated with more acute suicidality: PT and PD. Subsequent research endeavors should aim to empirically test the validity of these hypotheses.

Inadequate coping may lead to “white noise”. We are convinced that inadequate coping (IC) needs to be considered a serious type of suicidality; professionals should to take a deep and hard look at underlying motivation of the entrapment and suffering, being aware of counter transference playing a role in the assessment. Inadequate coping should never be a reason to justify withholding care. It probably indicates that the extreme boundaries of care that can be delivered are reached. It is preferable that mental health care professionals convert perceived impotence into a targeted search for alternatives, in joint cooperation with other parties, not least with the patient themselves.

Certain individuals might feign suicidal intentions without actually experiencing underlying suicidality [37]. They may adopt this pretense as a means of leveraging the threat of suicide for manipulative purposes, a form of coercion, rather than genuine emotional distress. This should be differentiated from the concept of 'blackmail' as an insufficient coping mechanism related to suicidal tendencies [38]. It is possible that this subset potentially intersects, though perhaps unjustifiably, with the notion of 'inadequate coping” (IC). It might be valuable to establish criteria that allow for withholding the label of 'suicidal' even when behaviour is presented as such. In cases of uncertainty, utmost caution must be exercised, and the term 'blackmail' should be used judiciously in the context of suicidality.

## Conclusions

Outcomes of this study suggest that clinicians who are asked to allocate cases of entrapment to subtypes of suicidality as described in the (hypothetical) 4-type of entrapment model for suicidality ((h)4ME) using the SUICIDE-2 instrument, show high interrater agreement and recognise different subtypes of suicidality.

## Data Availability

The datasets used and analysed during the current study are available from the corresponding author on reasonable request.
